# Unravelling a clinical role of peripheral blood leukemia stem cells at diagnosis in chronic myeloid leukemia patients: Final results of prospective FLOWERS study

**DOI:** 10.1002/cncr.70122

**Published:** 2025-10-15

**Authors:** Anna Sicuranza, Paola Pacelli, Adele Santoni, Elisabetta Abruzzese, Daniele Cattaneo, Alessandra Iurlo, Luigiana Luciano, Sara Galimberti, Valentina Giai, Olga Mulas, Giovanni Caocci, Federica Sorà, Isabella Capodanno, Monica Crugnola, Antonella Gozzini, Sabina Russo, Mario Annunziata, Claudio Fozza, Alessandra Cartocci, Sara Fredducci, Emanuele Pacini, Anna Marina Liberati, Marzia Defina, Elena Bestoso, Cristina Marzano, Dania Tocci, Teresa Miracapillo, Camilla Turriziani, Katia Peccia, Donatella Raspadori, Monica Bocchia

**Affiliations:** ^1^ Hematology Unit University of Siena Azienda Ospedaliera Universitaria Senese Siena Italy; ^2^ Sant'Eugenio Hospital Tor Vergata University Rome Italy; ^3^ Hematology Division Foundation IRCCS Ca' Granda Ospedale Maggiore Policlinico Milan Italy; ^4^ Hematology‐Department of Clinical Medicine and Surgery Federico II University Naples Italy; ^5^ Section of Hematology Department of Clinical and Experimental Medicine University of Pisa Pisa Italy; ^6^ Division of Hematology Città Della Salute e Della Scienza Turin Italy; ^7^ Department of Medical Sciences and Public Health University of Cagliari Cagliari Italy; ^8^ Dipartimento di Scienze di Laboratorio ed Ematologiche Fondazione Policlinico Universitario "A. Gemelli" IRCCS Rome Italy; ^9^ SOC Ematologia Azienda USL‐IRCSS di Reggio Emilia Reggio Emilia Italy; ^10^ Ematologia e Centro BMT Azienda Ospedaliero‐Universitaria di Parma Parma Italy; ^11^ Hematology Unit AOU Careggi University of Florence Florence Italy; ^12^ Hematology University of Messina Messina Italy; ^13^ Hematology Unit Hospital 'Antonio Cardarelli' Naples Italy; ^14^ Department of Medical, Surgical and Experimental Sciences University of Sassari Sassari Italy; ^15^ Department of Medical Sciences, Surgery and Neurosciences University of Siena Siena Italy; ^16^ Università degli studi di Perugia‐Struttura Complessa di Oncoematologia Terni Italy

**Keywords:** CD26, chronic myeloid leukemia, flow cytometry, leukemia stem cells, tyrosine kinase inhibitors

## Abstract

**Background:**

The authors previously demonstrated that in peripheral blood (PB) of chronic myeloid leukemia (CML), patients’ leukemia stem cells (LSCs) CD26+ are detectable by flow cytometry at diagnosis, during tyrosine kinase inhibitor (TKI) therapy, and during treatment‐free remission.

**Methods:**

This study presents results of a prospective multicenter study including 242 newly diagnosed CML patients monitored for PB CD26+ leukemic stem cells (LSCs) quantification from diagnosis up to 24 months of TKI treatment.

**Results:**

The bulk of CD26+ LSCs at diagnosis varied between patients with a median value of 7.14 cells/µL. During TKI treatment, it has been observed their consistent and rapid reduction without statistical differences according to type of first‐line TKI. Instead, a significant correlation between a low amount of CD26+ LSCs at diagnosis and an optimal molecular response at 3, 12, and 24 months was documented (*p* = .03, *p* = .004, and *p* = .009, respectively). Three tertiles of CD26+ LSCs correlating to molecular response were identified: <3.21 cells/µL; between 3.21 and 19.21 cells/µL; and >19.21 cells/µL. The incidence of patients with optimal response was higher in the first CD26+ LSCs tertile respect to the third one (*p* = .027, *p* = .015, and *p* = .079, respectively) at all time points (3, 12 and 24 months).

**Conclusions:**

This study demonstrated a correlation between the amount of CD26+ LSCs at diagnosis and the molecular response, suggesting that the number of CD26+ LSCs at diagnosis could represent an additional tool for predicting TKI response.

## Abstract 2

This prospective FLOWERS study demonstrated that a lower amount of circulating peripheral blood CD26+ leukemic stem cells (LSCs) at diagnosis in chronic myeloid leukemia patients is significantly associated with optimal molecular responses to tyrosine kinase inhibitor therapy. These findings support flow cytometry CD26+ LSCs measurement at diagnosis as a promising predictive biomarker for early risk stratification and treatment response prediction.

## INTRODUCTION

Chronic myeloid leukemia (CML) represents one of the most successfully treated hematological diseases. The benefits of tyrosine kinase inhibitors (TKIs) offer the possibility, for CML patients achieving a stable deep molecular remission (DMR), to attempt a discontinuation of therapy, yet maintaining a condition of treatment‐free remission (TFR).^1^, ^2^, ^3^ However, the persistence of leukemic stem cells (LSCs), intrinsically resistant to TKIs and potentially “invisible” to standard quantitative reverse transcription polymerase chain reaction (qRT‐PCR) BCR::ABL1 analysis, appears to prevent this state, causing a disease relapse.^4^, ^5^, ^6^ Survival of CML LSCs may be the consequence of activation of several BCR::ABL1 independent pathways.^7^ One mechanism of TKI resistance was suggested by showing in vitro that in low‐oxygen CML stem cell potential is maintained, and LSCs are capable of regenerating BCR::ABL1‐expressing/‐dependent progeny, although BCR::ABL1 protein is suppressed.^7^ Therefore, TKI‐resistant CML LSCs seem to be likely BCR::ABL1 transcript‐positive but unable to synthesize the active protein. As such, qRT‐PCR, the most sensitive assay to monitor disease status in CML patients, could be unsuitable to quantify residual quiescent CML LSCs that are transcriptionally silent. In the last decade, several studies investigated CML LSCs in bone marrow and peripheral blood (PB) samples, exploring their specific features and how they are involved in CML progression and in TKIs resistance.^8^, ^9^, ^10^, ^11^ CML LSCs have been demonstrated to reside within the CD34+/CD38−/Lin− fraction and to have an aberrant expression of cell surface molecules such as CD93, CD25, IL1RAP, and CD26 (dipeptidyl peptidase‐IV [DPP4]). The latter represents the most specific CML LSCs marker, discriminating these cells to the LSCs of other hematological diseases and also to the normal hematopoietic stem cells (HSCs).^8^, ^9^, ^10^, ^11^, ^12^

Our research group first demonstrated by a standardized flow cytometry method the presence of PB CD26+ LSCs in 100% of chronic phase (CP) CML patients at diagnosis, and a cross‐sectional study during TKIs treatment and TFR confirmed the persistence of circulating CD26+ LSCs with quite variable values between CML patients.^11^, ^13^ Additionally, in a recent multicenter prospective study, the behavior of PB CD26+ LSCs has been monitored in 109 CML patients suitable for TFR attempt, from the time of TKI discontinuation up to 12 months or until disease relapse, if any. Data showed that at the time of TKI withdrawal, 56% of CML patients studied still harbored a low, yet detectable amount of PB CD26+ LSCs independently to the previous TKI treatment. We also observed that the persistence of PB CD26+ LSCs did not correlate with the incidence of relapse, suggesting that other factors, such as an immune surveillance, could play a role in CML control at this stage of the disease. On the other hand, even patients with undetectable CD26+ LSCs at time of discontinuation could fail TFR.^14^ These results appeared controversial and induced us to further explore the role of CD26+ LSCs in the development of CML.

Albeit detectable in all CP CML patients at diagnosis, the amount of CD26+ LSCs is highly variable between patients. Thus, we conceived a multicentric prospective study with the aim to investigate LSCs behavior from diagnosis until 24 months of TKI treatment correlating the bulk of CD26+ LSCs at diagnosis with clinical features, molecular response, and disease progression.

## MATERIALS AND METHODS

### Patients cohort

After ethical committee approval, all consecutive patients diagnosed with CP CML referring to 15 participating hematology units, were enrolled in the study. After completing diagnostic assessment, patients started any first line‐approved TKIs (imatinib, nilotinib, or dasatinib) according to physician decision. CML patients were locally monitored for the entire study duration and subsequent follow‐up per standard guidelines. Clinical and molecular data were recorded on the patient's chart until collection and analysis. Before starting TKI therapy, patients gave written informed consent to participate in the study, in accordance with the Declaration of Helsinki.

### Flow cytometry PB CD26+ LSCs detection and BCR::ABL1 monitoring

Each enrolled CML patient was studied centrally in Siena for the presence of PB CD26+ LSCs at time 0 (baseline), after 3, 12, and 24 months of treatment, according to a standardized flow cytometry method developed in our laboratory. Six milliliters of PB were collected in EDTA and sent within 24 hours to the Siena hematology laboratory. The cellular fraction CD34+/CD38–/CD26+ was detected by using a multicolor flow cytometry instrument (BD FACSCanto II) and a BD FACSDiva software (BD Biosciences) with a protocol that implied adjustments of fluorescence‐activated cell sorting parameters, using the CS&T System (BD Biosciences), to keep constant the instrument performance by correcting wear of lasers and fluidic instability. The gating strategy is depicted in Figure S1. The median absolute number of CD26+ cells/μL has been calculated as follows: [No. WBCs/μL) × (% of CD34+/CD38−/CD26+ on CD45+ cells)].

The quantitative evaluation of BCR::ABL1 transcript has been performed at each established time point by each hematology center according to European Leukemia Net (ELN) guidelines^15^ and to the Italian molecular laboratory net for CML (Labnet CML).

### Statistical analysis

Descriptive statistics were performed by summarizing qualitative variables using absolute frequencies and percentages, while quantitative variables were described using the median, interquartile range (IQR), and minimum‐maximum range. Kruskal–Wallis test was performed to evaluate the difference of CD26+ LSCs at diagnosis between Sokal, the European Treatment and Outcome Study (EUTOS), and EUTOS‐long‐term survival (ELTS) scores. Post hoc analysis was performed and multiple Mann‐Whitney tests with false discovery rate correction were performed. Mann‐Whitney test was performed to compare CD26+ LSCs at diagnosis with molecular response at 3 months (BCR::ABL1 <10%) and at 12 and 24 months (BCR::ABL1 <0.1%). To establish cut offs for CD26+ LSCs at diagnosis, tertiles were estimated, and the associations with BCR::ABL1 <10% and BCR::ABL1 <0.1% were evaluated by χ^2^ test. A *p* value <.05 was considered statistically significant. All the analyses were performed by R version 4.3.1.

## RESULTS

### Patients cohort clinical data

Between January 2018 and December 2022, 242 CP‐CML patients at diagnosis were enrolled in this prospective noninterventional study. A total of 143 patients (59%) were males and 99 (41%) were females. At diagnosis, the median age in the whole cohort was 60 years (range, 18–90 years). Sokal score was high in 38 of 242 (16%) patients, intermediate in 87 of 242 (36%), low in 106 of 242 (44%), and not available in 11 of 242 (4%), whereas EUTOS score was high in 20 of 242 patients (8.2%), low in 202 of 242 (83.6%), and unknown in 20 of 242 (8.2%) patients. ELTS score was high in 32 of 242 (14%), intermediate in 85 of 242 (35%), low in 114 of 242 (47%) patients, and not available in 11 of 242 (4%), respectively.

The most frequent BCR::ABL1 transcript was b3a2 in 140 in 242 (58%) patients. Additional cytogenetic abnormalities were reported in only 13 of 242 patients (5.3%). Overall, 132 (54%) patients started treatment with imatinib, 72 (30%) with nilotinib, and 38 (16%) with dasatinib. It should be noted that 95 of 242 (39%) patients received cytoreductive therapy (hydroxyurea) before TKIs. Complete cytogenetic response was achieved at a median time of 4 months (range, 3–13 months). After a median time of observation of 66 months, 84 of 242 (35%) patients switched TKI treatment: 36 of 84 (43%) patients after failure or resistance and 48 of 84 (57%) patients due to intolerance. All clinical and hematological patients’ characteristics are described in Table 1.

**TABLE 1:** Patient’s characteristics.

	Whole cohort (*n* = 242)	Imatinib (*n* = 132)	Nilotinib (*n* = 72)	Dasatinib (*n* = 38)
Median age at diagnosis, years (range)	60 (18–90)	68 (18–90)	48 (18–85)	50 (30–79)
Sex, No. (%)				
Male	143 (59)	83 (63)	36 (50)	24 (63)
Female	99 (41)	49 (37)	36 (50)	14 (37)
Sokal score, No. (%)				
High	38 (16)	21 (16)	9 (13)	8 (21)
Intermediate	87 (36)	58 (44)	16 (22)	13 (34)
Low	106 (44)	46 (35)	45 (62)	15 (40)
Unknown	11 (4)	7 (5)	2 (3)	2 (5)
EUTOS score, No. (%)				
High	20 (8.2)	12 (12)	5 (7)	3 (7.9)
Low	202 (83.6)	109 (80)	62 (86)	31 (81.6)
Unknown	20 (8.2)	11 (8)	5 (7)	4 (10.5)
ELTS score, No. (%)				
High	32 (14)	19 (14.5)	3 (4)	10 (26)
Intermediate	85 (35)	43 (32.5)	22 (31)	20 (53)
Low	114 (47)	63 (48)	45 (62)	6 (16)
Unknown	11 (4)	7 (5)	2 (3)	2 (5)
BCR::ABL1 transcript, No. (%)				
b2a2	77 (32)	81 (61)	42 (58)	17 (45)
b3a2	140 (58)	40 (30)	22 (31)	15 (39)
b2a2/b3a2	11 (4)	4 (3)	5 (7)	2 (5)
Unknown	14 (6)	7 (6)	3 (4)	4 (11)
Additional cytogenetic abnormalities, No. (%)	13 (5.4)	5 (3.7)	3 (4)	5 (13)
Complete cytogenetic response (months)	4 (3–13)	4 (3–13)	4 (3–12)	4 (3–13)
Molecular response after starting therapy (BCR::ABL1%) (IQR)				
3 months	0.88 (0.15–4)	1.66 (0.47–8.3)	0.19 (0.08–1.00)	0.46 (0.09–3.44)
12 months	0.02 (0.006–0.17)	0.04 (0.008–0.23)	0.01 (0.003–0.06)	0.03 (0.01–0.21)
24 months	0.008 (0.002–0.03)	0.009 (0.002–0.04)	0.005 (0–0.01)	0.01 (0.002–0.04)
Cytoreductive therapy before TKI, No. (%)				
Yes	95 (39)	43 (32.5)	30 (42)	22 (58)
No	147 (61)	89 (67.5)	42 (58)	16 (42)
CD26+ LSCs/µL (IQR)				
Diagnosis	7.14 (2.18–33.26)	5.53 (1.79–20.14)	1.98 (2.44–61.24)	13.27 (3.06–44.57)
3 months	0.01 (0–0.03)	0.01 (0–0.04)	0.01 (0–0.03)	0.01 (0–0.03)
12 months	0.01 (0–0.03)	0.01 (0–0.002)	0.01 (0–0.04)	0.01 (0–0.02)
24 months	0.007 (0–0.02)	0.01 (0.005–0.05)	0.02 (0.002–0.06)	0.02 (0–0.02)
Switch TKI, No. (%)				
Yes	84 (35)	47 (36)	21 (29)	16 (42)
No	158 (65)	85 (64)	51 (71)	22 (58)
Reason for TKI switch, No. (%)				
Resistance	36 (43)	24 (51)	6 (28.6)	6 (37.5)
Intolerance	48 (57)	23 (49)	15 (71.4)	10 (62.5)

Abbreviations: ELTS, EUTOS‐long‐term survival; EUTOS, European Treatment and Outcome Study; IQR, interquartile range; TKI, tyrosine kinase inhibitor.

### CD26+ LSCs and BCR::ABL1 transcript monitoring from diagnosis and during TKI treatment

CD26+ leukemic stem cells (LSCs) were measured at diagnosis, as well as at 3 and 12 months of TKI treatment in all 242 CML patients (100%), and in 201 out of 242 patients (83%) at 24 months of TKI therapy. The median of CD26+ LSCs detected at diagnosis in the whole cohort of 242 CP‐CML patients was 7.14 cells/µL (range, 0.01–698.74 cells/µL; interquartile range [IQR], 2.18–33.26 cells/µL) without statistical differences according to TKI treatment: imatinib cohort median value of 5.53 cells/µL (IQR, 1.79–20.14 cells/µL); nilotinib cohort median value of 11.98 cells/µL (IQR, 2.44–61.24 cells/µL), and dasatinib cohort median value of 13.27 cells/µL (IQR, 3.06–44.57 cells/µL) (*p* = .05).

Regarding PB CD26+ LSCs behavior in the whole cohort during TKI treatment, we observed a consistent and rapid reduction with median values of 0.01 cells/µL (IQR, 0–0.03 cells/µL), 0.01 cells/µL (IQR, 0–0.03 cells/µL), and 0.007 cells/µL (IQR, 0–0.02 cells/µL) at 3, 12, and 24 months, respectively. No statistically significant differences in terms of CD26+ LSCs log‐reduction were noted according to the type of TKI treatment at any time points evaluated. Indeed, at 3 months we measured a median of 0.01 cells/µL (IQR, 0–0.04 cells/µL) in the imatinib group, 0.01 cells/µL (IQR, 0–0.03 cells/µL) in the nilotinib group, and 0.01 cells/µL (IQR, 0–0.03 cells/µL) in the dasatinib group (*p* = .216). Similar values of CD26+ LSCs were detected at 12 months and 24 months of treatment, with again, no differences between TKIs (Table 1).

With respect to molecular response in the whole cohort, the median value of BCR::ABL1 transcript was 0.88% (IQR, 0.15%–4.0%) at 3 months, 0.02% (IQR, 0.006%–0.17%) at 12 months, and 0.008% (IQR, 0.002%–0.03%) at 24 months of TKI treatment. The molecular response according to time and type of TKI treatment is detailed in Table 1.

### CD26+ LSCs bulk at diagnosis and correlation with clinical features and disease response

**FIGURE 1:**
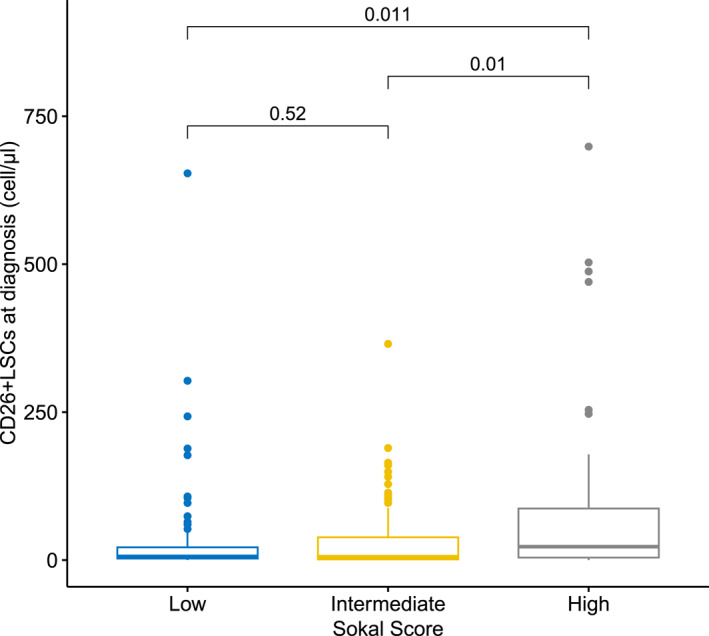
CD26+ LSCs bulk at diagnosis and correlation with Sokal score. A significant correlation between the bulk of CD26+ LSCs at diagnosis and Sokal score was documented (*p* = .018). CD26+ LSCs were significantly higher in the high Sokal risk group compared to the intermediate or low‐risk groups (22.65 cells/µL vs. 5.60 cells/µL vs. 6.16 cells/µL). LSCs indicate leukemia stem cells.

As clinical parameters, we chose to evaluate Sokal, EUTOS, and ELTS scores. Interestingly, a significant correlation between the bulk of CD26+ LSCs at diagnosis and Sokal score was documented (*p* = .018) with levels of CD26+ LSCs significantly higher in the high Sokal risk group compared to the intermediate or low risk groups (22.65 cells/µL vs. 5.60 cells/µL vs. 6.16 cells/µL) (Figure 1). Instead, no correlation was found between the bulk of CD26+ LSCs and low or high EUTOS score (*p* = .109) and high, intermediate, and low ELTS score (*p* = .224).

**FIGURE 2:**
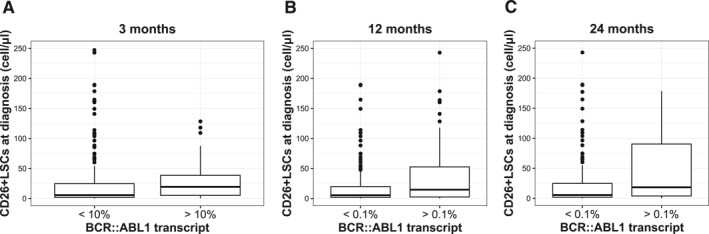
CD26+ LSCs bulk at diagnosis and correlation with molecular response. A lower amount of CD26+ LSCs at diagnosis was correlated with an optimal molecular response at 3, 12, and 24 months of TKI treatment. (A) CML patients with an optimal molecular response at 3 months (BCR::ABL1<10%) showed a median CD26+ LSCs at diagnosis of 6.21 cells/µL (IQR, 1.79–1.50 cells/µL) whereas suboptimal responders patients had 19.87 cells/µL at diagnosis (IQR, 5.37–39.81 cells/µL) (*p* = .03). (B) At 12 months, CML patients with BCR::ABL1 <0.1% showed a median amount of CD26+ LSCs of 5.50 cells/µL at diagnosis (IQR, 1.81–22.64 cells/µL) whereas suboptimal responders had a median of 16.87 cells/µL (IQR, 2.82–71.77 cells/µL) (*p* = .004). (C) At 24 months, CML patients with optimal molecular response had a median CD26+ LSCs value at diagnosis of 6.05 cells/µL (IQR, 1.79–29.90 cells/µL) compared to suboptimal responders showing a median value of 20.52 cells/µL (IQR, 4.24–106.91 cells/µL) (*p* = .009). CML indicates chronic myeloid leukemia; IQR, interquartile range; LSCs, leukemia stem cells; TKI, tyrosine kinase inhibitor.

Evaluating the amount of CD26+ LSCs at diagnosis in the whole cohort of 242 patients and subsequent molecular response, we observed that an optimal molecular response at 3, 12, and 24 months (considering BCR::ABL1 transcript <10% at 3 months and BCR::ABL1 transcript <0.1% at 12 and 24 months) was always correlated to a lower amount of CD26+ LSCs. Specifically, CML patients with an optimal molecular response at 3 months showed a median CD26+ LSCs at diagnosis of 6.21 cells/µL (IQR, 1.79–31.50 cells/µL) whereas suboptimal responders patients (BCR::ABL1 transcript >10%) had a median of 19.87 cells/µL at diagnosis (IQR, 5.37–39.81 cells/µL) (*p* = .03) (Figure 2A); at 12 months, CML patients with BCR::ABL1 <0.1% showed a median amount of CD26+ LSCs of 5.50 cells/µL at diagnosis (IQR, 1.81–22.64 cells/µL) whereas suboptimal responders (BCR::ABL1 transcript >0.1%) had a median of 16.87 cells/µL (IQR, 2.82–71.77 cells/µL) (*p* = .004) (Figure 2B). At 24 months of TKI treatment, CML patients with optimal molecular response had a median CD26+ LSCs value at diagnosis of 6.05 cells/µL (IQR, 1.79–29.90 cells/µL) compared with suboptimal responders, with BCR::ABL1 transcript >0.1%, showing a median value of 20.52 cells/µL (IQR, 4.24–106.91 cells/µL) (*p* = .009) (Figure 2C).

To further confirm that the correlation between the amount of CD26+ LSCs with Sokal score and molecular response is “CD26+ LSCs‐specific” and not just related to the broad CD34+CD38– stem cell compartment, we performed a separate analysis of the CD34+CD38– population excluding the CD34+CD38–CD26+ subset. As expected, no statistically significant correlation was found (experimental details in Table S1).

Even when considering CML patients according to the type of TKI, comparing imatinib and 2G‐TKIs, (nilotinib + dasatinib), we observed a statistical correlation between a lower amount of CD26+ LSCs at diagnosis and an optimal molecular response at 3, 12, and 24 months of treatment for imatinib group (*p* = .018, *p* = .019, and *p* = .004, respectively) and at 3 and 12 months for 2G‐TKIs group (*p* = .030 and *p* = .050, respectively). No statistical correlation between optimal molecular response and low amount of CD26+ LSCs for 2G‐TKIs group was found after 24 months of treatment (*p* = .553).

Moreover, looking at CML patients who failed molecular response after first line TKI and thus switched treatment (36 of 242, 15%), we observed that these patients had a significantly higher median CD26+ LSCs at diagnosis, accounting for 14.59 cells/µL (IQR, 3.76–46 cells/µL) compared with a median CD26+ LSCs value of 5.82 cells/µL (IQR, 2.35–26.70 cells/µL) in the no‐switch group (*p* = .034).

A total of 46 of 242 (19%) CML patients attempted a discontinuation of TKI treatment with a median value of CD26+ LSCs at diagnosis of 8.67 cells/µL. Of these, 20 of 46 (43.5%) patients lost TFR, and 26 of 46 (56.5%) patients maintained TFR. Median values of CD26+ LSCs at diagnosis were 9.10 cells/µL and 8.25 cells/µL, respectively, without statistical differences.

### CD26+ LSCs threshold at diagnosis predictive of molecular response

To further analyze the relationship between the burden of CD26+ LSCs at diagnosis and BCR::ABL1 transcript reduction after TKI treatment to identify cutoffs of CD26+ LSCs suggestive/predictive of molecular response, the absolute number of CD26+ LSC at diagnosis was divided in tertiles. Three ranges of CD26+ LSCs were obtained: <3.21 cells/µL (1° tertile); between 3.21 and 19.21 cells/µL (2° tertile); and >19.21 cells/µL (3° tertile). We observed an association between CD26+ LSCs tertile and the rate of optimal molecular response at a given time point. In particular, considering the molecular response at 3 months, the incidence of CML patients with BCR::ABL1 <10% was 93.5% in the first CD26+ LSCs tertile, whereas it was 78.8% in the third tertile (*p* = .027). At 12 months, the incidence of optimal response in the first tertile was 78.5% and 62.8% in the third tertile (*p* = .015). At 24 months, the two incidences were 90.8% and 77.9%, respectively (*p* = .079). The second tertile has an intermediate incidence of optimal response (Table S2). A similar analysis, considering CD26+ LSC tertiles at diagnosis according to the type of TKI treatment, was not performed due to the different sample size between treatment groups.

## DISCUSSION

Following several findings that PB CD26+ LSCs are present at diagnosis in all CP CML patients and persist in a significant number of them during TKI treatment and TFR, this study prospectively unravels the fate of this peculiar CML cellular marker and provides the first evidence of its predictive value for disease response. First, we confirmed that in all 242 patients PB CD26+ LSCs were measurable at diagnosis albeit with a great value variability between patients ranging from 0.01 to 698 cells/µL (median, 7.14 cells/µL). However, we showed that PB CD26+ LSCs rapidly and deeply reduce during TKI treatment remaining detectable with fluctuating similar values from then on. No differences in terms of degree of reduction of PB CD26+ LSCs were found when comparing imatinib, nilotinib, and dasatinib TKI treatment cohorts. As previously observed,^11^ even in this prospective study, we confirmed no statistical correlation between the number of residual PB CD26+ LSCs and the copies of BCR::ABL1 transcript at any time of treatment. Although they may well represent a disease reservoir, the slope of reduction and the residual number of PB CD26+ LSCs appear not suitable for monitoring a sort of “stem cell response” and cannot be considered a surrogate of molecular response.

Yet, the variable bulk of this specific stem cell compartment found at diagnosis could still somehow influence disease biology and response to treatment.

Thus, we first examined the correlation between the absolute number of PB CD26+ LSCs at diagnosis and the Sokal score. As documented by Figure 1, CD26+ LSCs were significantly higher in the high Sokal risk group, suggesting a possible negative prognostic role of CD26+ LSCs. On the contrary, no statistical correlation between CD26+ LSCs at diagnosis and EUTOS and ELTS scores were found, the latter as a possible consequence of different prognostic goals, different types of parameters chosen, and different weight given to the same parameters by these three prognostic scores.

To further investigate if the different amount of CD26+ LSCs at diagnosis can be predictive of short‐ and long‐term response, their absolute number has been correlated with the degree of molecular response obtained at 3, 12, and 24 months of TKI treatment according to ELN guidelines.

Interestingly, a statistically significant correlation between a low amount of CD26+ LSCs at diagnosis and an optimal molecular response was documented at any time point; in contrast a significantly higher median CD26+ LSCs at diagnosis was observed in those patients who had to switch TKI due to treatment failure. Based on these results, we tried to identify a specific threshold of CD26+ LSCs at diagnosis that was able to discriminate between CML patients achieving an optimal or suboptimal response after 3, 12, and 24 months of therapy. Statistical analysis on our cohort provided three cutoff values (tertiles): below 3.21 cells/µL, between 3.21 and 19.21 cells/µL, and above 19.21 cells/µL. The lowest and highest tertiles of CD26+ LSC significantly differentiate CML patients achieving or not an optimal molecular response at 3 and 12 months, respectively. At 24 months, the incidence of optimal response is still directly or inversely correlated to the first and third CD26+ LSCs tertile, yet without statistical significance, it is probably due to a reduced number of patients achieving this time point. Because CD26+ LSCs belongs to the broader CD34+CD38– stem cell compartment, one would inquire if it is the bulk of these general stem cells found at diagnosis, and not the specific CD26+ LSCs subset that influences the signature of CML and the response to treatment. To clarify this issue, we performed a separate analysis of the CD34+CD38–population excluding the CD34+CD38–CD26+ subset and no correlation with SOKAL score and molecular response was found, thus further confirming that is the amount of CD26+ LSCs that counts.

This multicenter prospective study, including a not negligible number of CML patients, links, for the first time, the “bulk” of circulating CML‐specific LSCs at the diagnosis with the subsequent attainment of molecular response. Even if we identified CD26+ LSCs tertiles values correlating with the probability of achieving an optimal response at any of the main treatment check points considered in the routinely management of CML, we are aware that one limitation of our study, due to the great variability of PB CD26+ LSCs number found at diagnosis, is the lack of a single predictive‐of‐response CD26+ LSCs threshold value that could be more easily included in a clinical strategy of CML management. The evidence of a prognostic role of the burden of CD26+ LSCs at diagnosis could appear in conflict with the rapid and deep reduction of PB LSCs documented in all CML patients and with any first line TKI treatment. Yet, it has to be considered that most CML patients harbor residual PB CD26+ LSCs even when BCR::ABL1 transcript is undetectable and/or during TFR, thus arguing that other factors besides their absolute number may influence TKI response and ultimately disease course. In this regard, preliminary data suggest that the phenotype at diagnosis of CD26+ LSCs may be different between CML patients, particularly when considering quiescence properties (i.e., ability to survive in the low‐oxygen hematopoietic environment) and the level of expression of surface molecules interacting with the immune system such as PDL‐1 and CD47 (unpublished personal data). These biological differences in CD26+ LSCs compartment could partly explain why the persistence of residual staminal disease does not impair the attainment of a DMR and a stable TFR. Similarly, a peculiar CD26+ LSCs phenotype together with their absolute number at diagnosis could influence the degree of molecular response to TKI. Hopefully, both additional and deeper knowledge on the inner biologic feature of CD26+ LSCs, as well as a longer follow‐up, would help to strengthen the clinical role of these cells (i.e., bulk value at diagnosis and TFR rate?).

Although the exact significance of the presence and persistence of these unique cells in the natural history of CML is not yet fully understood, our data demonstrate a correlation between the amount of PB CD26+ LSCs at diagnosis and the molecular response. Given these results, flow cytometry measurement of the absolute number of PB CD26+ LSCs represents not only an easy and rapid diagnostic tool, it could also furnish additional information for predicting TKI response.

## AUTHOR CONTRIBUTIONS

**Anna Sicuranza**: Methodology; writing—original draft; writing—review and editing; data curation; and conceptualization. **Paola Pacelli**: Methodology; writing—original draft; writing—review and editing; and conceptualization. **Adele Santoni**: Investigation and writing—review and editing. **Elisabetta Abruzzese**: Investigation and writing—review and editing. **Daniele Cattaneo**: Investigation and writing—review and editing. **Alessandra Iurlo**: Investigation and writing—review and editing. **Luigiana Luciano**: Investigation and writing—review and editing. **Sara Galimberti**: Investigation and writing—review and editing. **Valentina Giai**: Investigation and writing—review and editing. **Olga Mulas**: Investigation and writing—review and editing. **Giovanni Caocci**: Investigation and writing—review and editing. **Federica Sora**: Investigation and writing—review and editing. **Isabella Capodanno**: Investigation and writing—review and editing. **Monica Crugnola**: Investigation and writing—review and editing. **Antonella Gozzini**: Investigation and writing—review and editing. **Sabina Russo**: Investigation and writing—review and editing. **Mario Annunziata**: Investigation and writing—review and editing. **Claudio Fozza**: Investigation and writing—review and editing. **Alessandra Cartocci**: Formal analysis; writing—review and editing; data curation; and software. **Sara Fredducci**: Investigation and writing—review and editing. **Emanuele Pacini**: Investigation and writing—review and editing. **Anna Marina Liberati**: Investigation and writing—review and editing. **Marzia Defina**: Investigation and writing—review and editing. **Elena Bestoso**: Methodology. **Cristina Marzano**: Methodology. **Dania Tocci**: Methodology. **Teresa Miracapillo**: Methodology. **Camilla Turriziani**: Methodology. **Katia Peccia**: Methodology. **Donatella Raspadori**: Methodology; supervision; and writing—review and editing. **Monica Bocchia**: Conceptualization; investigation; funding acquisition; writing—original draft; writing—review and editing; and supervision. All authors revised and approved the final manuscript.

## CONFLICT OF INTEREST STATEMENT

Elisabetta Abruzzese reports consulting fees from Pharma, Bristol Myers Squibb, GlaxoSmithKline, Incyte Corporation, Instituto Científico Pfizer, and Novartis. Monica Bocchia reports consulting fees from Incyte Corporation and Novartis; and travel fees from BeiGene USA, Inc. Monica Crugnola reports consulting fees from Novartis. Valentina Giai reports consulting fees from Novartis, Pfizer, and Sobi; and fees for expert witness testimony from Alexion Pharmaceuticals. The other authors declare no conflicts of interest.

## Supporting information

Supplementary Material

Figure S1

Table S1

Table S2

## Data Availability

The data that support the findings of this study are available in the supplementary material of this article

## References

[1] Hochhaus A , Baccarani M , Silver RT , et al. European LeukemiaNet 2020 recommendations for treating chronic myeloid leukemia. Leukemia. 2020;34(4):966‐984. doi:10.1038/s41375-020-0776-2 32127639 PMC7214240

[2] Baccarani M , Abruzzese E , Accurso V , et al. Managing chronic myeloid leukemia for treatment‐free remission: a proposal from the GIMEMA CML WP. Blood Adv. 2019;3(24):4280‐4290. doi:10.1182/bloodadvances.2019000865 31869412 PMC6929396

[3] Laganà A , Scalzulli E , Bisegna ML , et al. Treatment free remission (TFR) after second‐generation tyrosine kinase inhibitors (2G‐TKIs) treatment in chronic myeloid leukemia (CML): from feasibility to safety. Expert Opin Drug Saf. 2024;23(8):969‐979. doi:10.1080/14740338.2024.2368822 38873693

[4] Saifullah HH , Lucas CM . Treatment‐free remission in chronic myeloid leukemia: can we identify prognostic factors? Cancers (Basel). 2021;13(16):4175. doi:10.3390/cancers13164175 34439327 PMC8392063

[5] Chen Y , Zou J , Cheng F , Li W . Treatment‐free remission in chronic myeloid leukemia and new approaches by targeting leukemia stem cells. Front Oncol. 2021;11:769730. doi:10.3389/fonc.2021.769730 34778088 PMC8581243

[6] Mojtahedi H , Yazdanpanah N , Rezaei N . Chronic myeloid leukemia stem cells: targeting therapeutic implications. Stem Cell Res Ther. 2021;12(1):603. doi:10.1186/s13287-021-02659-1 34922630 PMC8684082

[7] Hamilton A , Helgason GV , Schemionek M , et al. Chronic myeloid leukemia stem cells are not dependent on Bcr‐Abl kinase activity for their survival. Blood. 2012;119(6):1501‐1510. doi:10.1182/blood-2010-12-326843 22184410 PMC3286213

[8] Herrmann H , Sadovnik I , Cerny‐Reiterer S , et al. Dipeptidylpeptidase IV (CD26) defines leukemic stem cells (LSC) in chronic myeloid leukemia. Blood. 2014;123(25):3951‐3962. doi:10.1182/blood-2013-10-536078 24778155

[9] Valent P , Sadovnik I , Ráčil Z , et al. DPPIV (CD26) as a novel stem cell marker in Ph+ chronic myeloid leukaemia. Eur J Clin Invest. 2014;44(12):1239‐1245. doi:10.1111/eci.12368 25371066

[10] Culen M , Borsky M , Nemethova V , et al. Quantitative assessment of the CD26+ leukemic stem cell compartment in chronic myeloid leukemia: patient‐subgroups, prognostic impact, and technical aspects. Oncotarget. 2016;7(22):33016‐33024. doi:10.18632/oncotarget.9108 27145281 PMC5078071

[11] Bocchia M , Sicuranza A , Abruzzese E , et al. Residual peripheral blood CD26^+^ leukemic stem cells in chronic myeloid leukemia patients during TKI therapy and during treatment‐free remission. Front Oncol. 2018;8:194. doi:10.3389/fonc.2018.00194 29900128 PMC5988870

[12] Sicuranza A , Raspadori D , Bocchia M . CD26/DPP‐4 in chronic myeloid leukemia. Cancers (Basel). 2022;14(4):891. doi:10.3390/cancers14040891 35205639 PMC8870104

[13] Raspadori D , Pacelli P , Sicuranza A , et al. Flow cytometry assessment of CD26^+^ leukemic stem cells in peripheral blood: a simple and rapid new diagnostic tool for chronic myeloid leukemia. Cytometry B Clin Cytom. 2019;96(4):294‐299. doi:10.1002/cyto.b.21764 30714299 PMC6767040

[14] Pacelli P , Santoni A , Sicuranza A , et al. Prospective monitoring of chronic myeloid leukemia patients from the time of TKI discontinuation: the fate of peripheral blood CD26^+^ leukemia stem cells. Front Pharmacol. 2023;14:1194712. doi:10.3389/fphar.2023.1194712 37305536 PMC10250640

[15] Cross NCP , Ernst T , Branford S , et al. European LeukemiaNet laboratory recommendations for the diagnosis and management of chronic myeloid leukemia. Leukemia. 2023;37(11):2150‐2167. doi:10.1038/s41375-023-02048-y 37794101 PMC10624636

