# Supplementary material for: Unravelling a clinical role of peripheral blood leukemia stem cells at diagnosis in chronic myeloid leukemia patients: Final results of prospective FLOWERS study
Source: Cancer. 2025 Oct 15;131(20):e70122. doi: 10.1002/cncr.70122 (PMC12526715; doi:10.1002/cncr.70122)
Supplement: Supplementary file 1 — Supplementary Material [file CNCR-131-e70122-s001.docx]

**Supplemental Figure Legend. Flow cytometry CD26+LSCs gating strategy in a representative CML patient at diagnosis.**

**a.** identification of viable cells by FSC and SSC properties; **b.** CD45 expression versus SSC; **c**. gate of CD34+ cells; **d**. CD34+CD38- fraction on CD34+ cells; **e**. negative control on CD34+CD38- fraction; **f**. CD26+ expression on CD34+CD38- gated cells. **g**. statistical panel showing all populations selected.
