# Supplementary figures and images for: Unravelling a clinical role of peripheral blood leukemia stem cells at diagnosis in chronic myeloid leukemia patients: Final results of prospective FLOWERS study
Source: Cancer. 2025 Oct 15;131(20):e70122. doi: 10.1002/cncr.70122 (PMC12526715; doi:10.1002/cncr.70122)

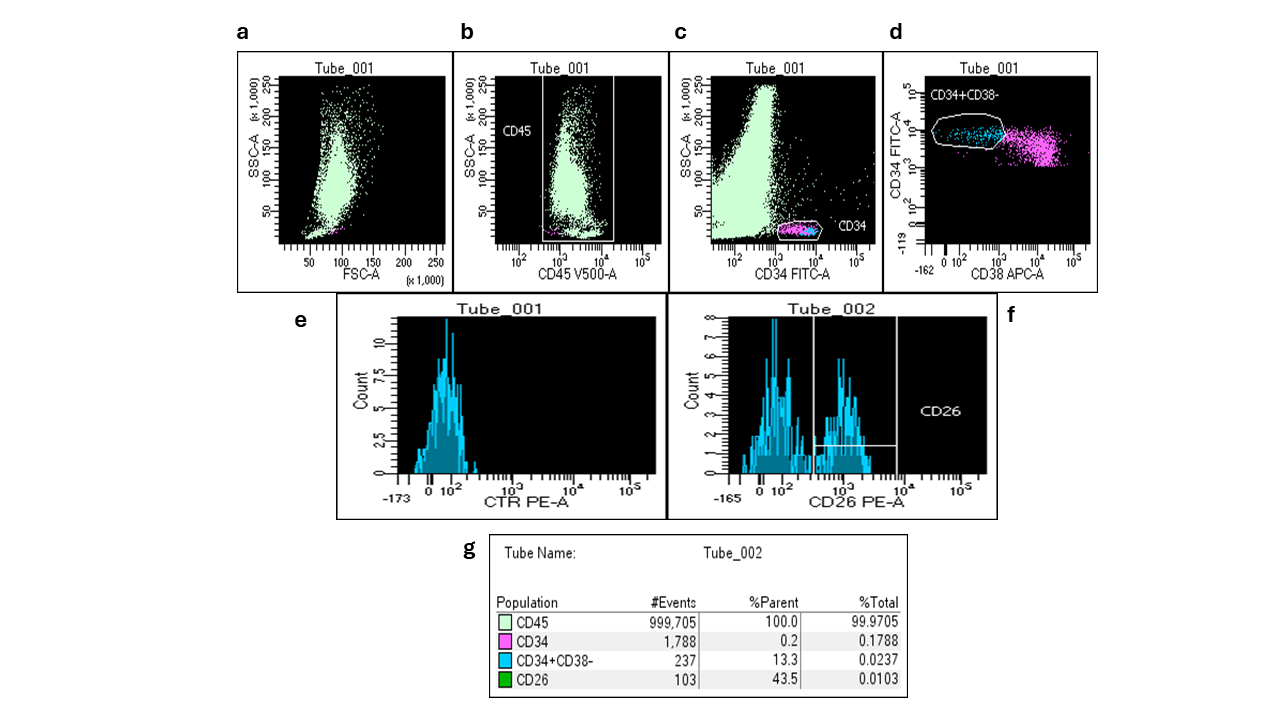

Supplement: Supplementary file 2 — Figure S1 [file CNCR-131-e70122-s002.tif]
