# Supplementary material for: Unravelling a clinical role of peripheral blood leukemia stem cells at diagnosis in chronic myeloid leukemia patients: Final results of prospective FLOWERS study
Source: Cancer. 2025 Oct 15;131(20):e70122. doi: 10.1002/cncr.70122 (PMC12526715; doi:10.1002/cncr.70122)
Supplement: Supplementary file 3 — Table S1 [file CNCR-131-e70122-s003.docx]

**Supplemental table 1 A-D. Correlation between CD26+LSCs at diagnosis and Sokal score (A); correlation between CD26+LSCs at diagnosis and molecular response (B-D).**

**A**

|  | **Sokal score**  **high** | **Sokal score**  **intermediate** | **Sokal score**  **low** | **p** |
| --- | --- | --- | --- | --- |
| **n** | 38 | 87 | 106 |  |
| **CD26 cells/µL at diagnosis (median)**  **[IQR]** | 22.65  [4.12, 87.12] | 5.60  [1.06, 38.51] | 6.16  [2.63, 21.56] | 0.018 |
| **CD34+CD38- total (median)**  **[IQR]** | 61.57  [14.08, 225.12] | 23.88  [5.91, 104.85] | 19.57  [8.46, 44.03] | 0.035 |
| **CD34+CD38- without CD26+ (median)**  **[IQR]** | 19.66  [2.95, 89.30] | 12.48  [3.82, 50.70] | 10.42  [4.02, 32.91] | 0.527 |
| **CD34+CD38-CD26+ (median)**  **[IQR]** | 49.15  [27.61, 67.68] | 35.05  [18.43, 58.96] | 33.75  [21.54, 57.42] | 0.204 |

| **Molecular response at 3 months** | **Optimal response (BCR::ABL1 <10%)** | **Suboptimal response (BCR::ABL1 >10%)** | **p** |
| --- | --- | --- | --- |
| **n** | 201 | 32 |  |
| **CD26 cells/µL at diagnosis (median)**  **[IQR]** | 6.21  [1.79, 31.50] | 19.87  [5.37, 39.81] | 0.030 |
| **CD34+CD38- total (median)**  **[IQR]** | 20.53  [7.17, 83.68] | 46.73  [19.19, 124.17] | 0.016 |
| **CD34+CD38- without CD26+ (median)**  **[IQR]** | 10.68  [3.90, 39.65] | 17.76  [5.05, 78.44] | 0.216 |
| **CD34+CD38-CD26+ (median)**  **[IQR]** | 34.07  [18.25, 60.22] | 45.95  [24.47, 63.45] | 0.207 |

**B**

| **Molecular response at 12 months** | **Optimal response (BCR::ABL1 <0.1%)** | **Suboptimal response (BCR::ABL1 >0.1%)** | **p** |
| --- | --- | --- | --- |
| **n** | 174 | 60 |  |
| **CD26 cells/µL at diagnosis (median)**  **[IQR]** | 5.50  [1.81, 22.64] | 16.87  [2.82, 71.77] | 0.004 |
| **CD34+CD38- total (median)**  **[IQR]** | 20.40  [7.25, 71.71] | 33.81  [13.67, 143.83] | 0.034 |
| **CD34+CD38- without CD26+ (median)**  **[IQR]** | 10.53  [3.84, 38.95] | 14.39  [4.47, 70.46] | 0.317 |
| **CD34+CD38-CD26+ (median)**  **[IQR]** | 32.76  [17.30, 51.53] | 49.52  [25.72, 70.74] | 0.004 |

**C**

| **Molecular response at 24 months** | **Optimal response (BCR::ABL1 <0.1%)** | **Suboptimal response (BCR::ABL1 >0.1%)** | **p** |
| --- | --- | --- | --- |
| **n** | 191 | 34 |  |
| **CD26 cells/µL at diagnosis (median)**  **[IQR]** | 6.06  [1.79, 29.90] | 20.52  [4.24, 106.91] | 0.009 |
| **CD34+CD38- total (median)**  **[IQR]** | 22.08  [7.33, 81.67] | 48.34  [10.33, 196.51] | 0.054 |
| **CD34+CD38- without CD26+ (median)**  **[IQR]** | 12.43  [3.85, 39.95] | 23.09  [5.15, 85.00] | 0.250 |
| **CD34+CD38-CD26+ (median)**  **[IQR]** | 32.68  [17.58, 57.69] | 48.58  [26.89, 67.46] | 0.037 |

**D**
