# Supplementary material for: Unravelling a clinical role of peripheral blood leukemia stem cells at diagnosis in chronic myeloid leukemia patients: Final results of prospective FLOWERS study
Source: Cancer. 2025 Oct 15;131(20):e70122. doi: 10.1002/cncr.70122 (PMC12526715; doi:10.1002/cncr.70122)
Supplement: Supplementary file 4 — Table S2 [file CNCR-131-e70122-s004.docx]

|  | CD26+LSCs at diagnosis  [0-3.21 cells/µl] | CD26+LSCs at diagnosis  [3.21-19.21 cells/µl] | CD26+LSCs at diagnosis  [>19.21 cells/µl] | p value |
| --- | --- | --- | --- | --- |
| Molecular response at 3 months (BCR::ABL1<10%) | 72 (93.5%) | 66 (86.8) | 63 (78.8%) | 0.027 |
| Molecular response at 3 months (BCR::ABL1>10%) | 5 (6.5%) | 10 (13.2%) | 17 (21.2%) |  |
| Molecular response at 12 months (BCR::ABL1<0.1%) | 62 (78.5%) | 63 (81.8%) | 49 (62.8%) | 0.015 |
| Molecular response at 12 months (BCR::ABL1>0.1%) | 17 (21.5%) | 14 (18.2%) | 29 (37.2%) |  |
| Molecular response at 24 months (BCR::ABL1<0.1%) | 69 (90.8%) | 62 (86.1%) | 60 (77.9%) | 0.079 |
| Molecular response at 24 months (BCR::ABL1>0.1%) | 7 (9.2%) | 10 (13.9%) | 17 (22.1%) |  |

**Supplemental table 2. Tertiles of CD26+LSCs at diagnosis and molecular response**
